# Alternative to prophylactic antibiotics for the treatment of recurrent urinary tract infections in women: multicentre, open label, randomised, non-inferiority trial

**DOI:** 10.1136/bmj-2021-0068229

**Published:** 2022-03-09

**Authors:** Chris Harding, Helen Mossop, Tara Homer, Thomas Chadwick, William King, Sonya Carnell, Jan Lecouturier, Alaa Abouhajar, Luke Vale, Gillian Watson, Rebecca Forbes, Stephanie Currer, Robert Pickard, Ian Eardley, Ian Pearce, Nikesh Thiruchelvam, Karen Guerrero, Katherine Walton, Zahid Hussain, Henry Lazarowicz, Ased Ali

**Affiliations:** 1 Department of Urology, Freeman Hospital, Newcastle upon Tyne, UK; 2 Population Health Sciences Institute, Newcastle University, Newcastle upon Tyne, UK; 3 Newcastle Clinical Trials Unit, Newcastle University, Newcastle upon Tyne, UK; 4 Translational and Clinical Research Institute, William Leech Building, The Medical School, Newcastle upon Tyne, UK; 5 Leeds Teaching Hospital Trust, Leeds, UK; 6 Manchester University Hospitals NHS Foundation Trust, Manchester, UK; 7 Addenbrooke’s Hospital, Cambridge, UK; 8 Queen Elizabeth University Hospital, Glasgow, UK; 9 Department of Microbiology, Freeman Hospital, Newcastle upon Tyne, UK; 10 Royal Oldham Hospital, Oldham, UK; 11 Liverpool University Hospitals NHS Foundation Trust, Liverpool, UK; 12 Pinderfields Hospital, Wakefield, UK

## Abstract

**Objective:**

To test and compare the efficacy of methenamine hippurate for prevention of recurrent urinary tract infections with the current standard prophylaxis of daily low dose antibiotics.

**Design:**

Multicentre, open label, randomised, non-inferiority trial.

**Setting:**

Eight centres in the UK, recruiting from June 2016 to June 2018.

**Participants:**

Women aged ≥18 years with recurrent urinary tract infections, requiring prophylactic treatment.

**Interventions:**

Random assignment (1:1, using permuted blocks of variable length via a web based system) to receive antibiotic prophylaxis or methenamine hippurate for 12 months. Treatment allocation was not masked and crossover between arms was allowed.

**Main outcome measure:**

Absolute difference in incidence of symptomatic, antibiotic treated, urinary tract infections during treatment. A patient and public involvement group predefined the non-inferiority margin as one episode of urinary tract infection per person year. Analyses performed in a modified intention-to-treat population comprised all participants observed for at least six months.

**Results:**

Participants were randomly assigned to antibiotic prophylaxis (n=120) or methenamine hippurate (n=120). The modified intention-to-treat analysis comprised 205 (85%) participants (antibiotics, n=102 (85%); methenamine hippurate, n=103 (86%)). Incidence of antibiotic treated urinary tract infections during the 12 month treatment period was 0.89 episodes per person year (95% confidence interval 0.65 to 1.12) in the antibiotics group and 1.38 (1.05 to 1.72) in the methenamine hippurate group, with an absolute difference of 0.49 (90% confidence interval 0.15 to 0.84) confirming non-inferiority. Adverse reactions were reported by 34/142 (24%) in the antibiotic group and 35/127 (28%) in the methenamine group and most reactions were mild.

**Conclusion:**

Non-antibiotic prophylactic treatment with methenamine hippurate might be appropriate for women with a history of recurrent episodes of urinary tract infections, informed by patient preferences and antibiotic stewardship initiatives, given the demonstration of non-inferiority to daily antibiotic prophylaxis seen in this trial.

**Trial registration:**

ISRCTN70219762.

**Figure:**
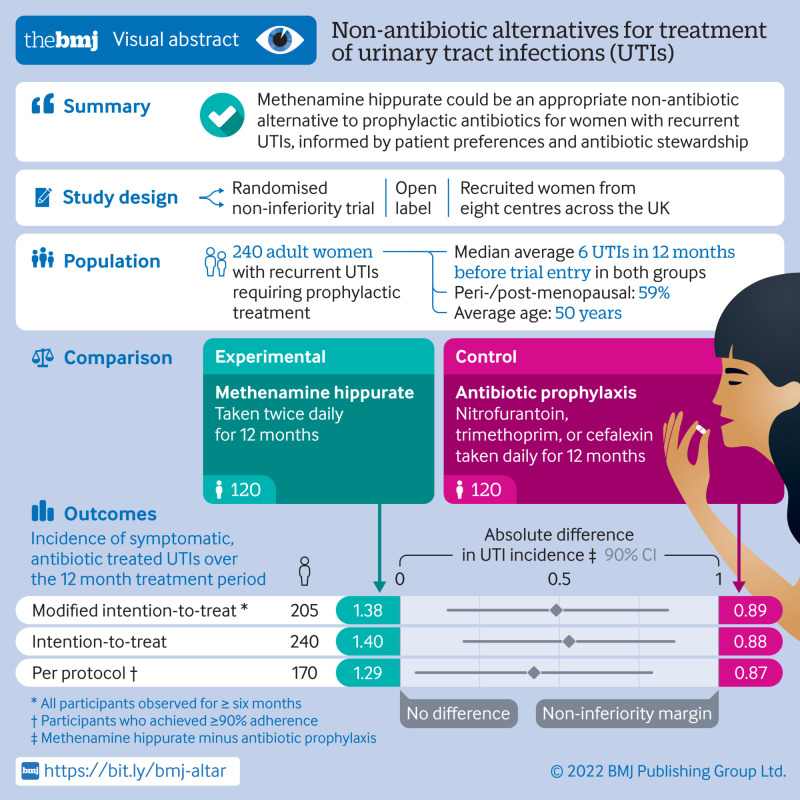


## Introduction

Recurrent urinary tract infection (UTI) is defined as repeated UTI with a frequency of at least two episodes in the preceding six months or three episodes in the past year.^1^ ^2^ Acute UTI is most often uncomplicated cystitis and occurs in 50-80% of women in the general population.^3^ About one in four women with one UTI episode will go on to develop frequent recurrences,^4^ representing a substantial global healthcare problem. An economic analysis from the US described UTI as accounting for more than 6.8 million consultations, 1.3 million emergency department visits, and 245 000 hospital admissions with an annual cost of more than US$2.4bn (£1.8bn; €2.1bn).^5^ National and international guidelines acknowledge the need for preventive strategies, and those from the UK, Europe, and US strongly recommend the use of daily, low dose antibiotics as the standard prophylactic treatment for recurrent UTI.^1^ ^2^ ^6^

The urgent need for demonstration of effective non-antibiotic treatments is underlined by the UK antimicrobial resistance strategy, which comments that “we are heading rapidly towards a world in which our antibiotics no longer work” and recommends a “strong focus on infection prevention.”^7^ One aim of this strategy is to reduce antimicrobial use in people by 15% before 2024; to achieve that, exploration of non-antibiotic preventive treatments in common conditions such as UTI is essential. Methenamine hippurate is one such non-antibiotic treatment, which is hydrolysed to formaldehyde in acidic environments such as the distal tubules of the kidney. Formaldehyde is bacteriocidal and works by denaturing bacterial proteins and nucleic acids.

Methenamine hippurate has been evaluated in previous Cochrane systematic reviews,^8^ which concluded that “methenamine hippurate may be effective for preventing UTI” but recognised the “need for further large well-conducted RCTs [randomised controlled trials] to clarify.” This study aimed to determine whether methenamine hippurate was an effective alternative to the standard treatment of low dose antibiotics for prophylaxis in women with recurrent UTI in a routine clinical setting. We tested the null hypotheses that methenamine hippurate was inferior to daily antibiotics for the prevention of recurrent UTI in women.

## Methods

### Study design

This pragmatic, multicentre, randomised, open label, non-inferiority trial compared clinical effectiveness of low dose antibiotic prophylaxis, the current standard treatment for recurrent UTI prevention, with the urinary antiseptic methenamine hippurate. The ALTAR trial (alternative to prophylactic antibiotics for the treatment of recurrent urinary tract infections in women) recruited women from secondary care urology and urogynaecology centres in the UK from June 2016 and incorporated a 12 month treatment period followed by a six month follow-up period. Recruitment was completed in June 2018 and the final follow-up visit took place in January 2020. The study protocol has been published elsewhere.^9^ ^10^

### Participants

Adult women aged 18 years and over with recurrent UTI who had decided, in conjunction with their responsible clinician, that prophylaxis was appropriate, were eligible for inclusion. Recurrent UTI was defined as at least three episodes of symptomatic UTI in the previous 12 months or at least two episodes in the past six months. We excluded women with correctable urinary tract abnormalities contributory to recurrent UTI (eg, urinary tract calculi) and those with neurogenic dysfunction of the lower urinary tract. Women already taking antibiotic prophylaxis or methenamine hippurate were allowed to take part, but a washout period of three months without preventive treatment was required before randomisation. All participants provided written informed consent.

### Randomisation and masking

Participants were randomly assigned (1:1) to receive antibiotic prophylaxis or methenamine hippurate. Permuted blocks of variable length (2/4/6/8) were used, stratified by menopausal status (pre-menopausal *v* peri-menopausal/post-menopausal) and UTI frequency in the preceding year (<4 *v* ≥4). Randomisation lists were generated by an individual not otherwise involved in the trial and administered centrally via a web based service. This pragmatic trial was designed to reflect contemporary practice, and there was no masking of participants, clinicians, or local research staff.

### Procedures

For participants assigned to antibiotic prophylaxis, the drug used was chosen from nitrofurantoin (50 or 100 mg), trimethoprim (100 mg), or cefalexin (250 mg) given orally once daily, depending on previous urine culture results and individuals’ history of allergy or intolerance. Methenamine hippurate was prescribed as a twice daily oral dose (1 g). Participants were allowed to switch between antibiotic drugs or between treatment strategies, however, the need to adhere to the allocated intervention was emphasised. Participants experiencing symptomatic UTI episodes were advised to seek discrete treatment courses of antibiotics in their usual way, typically via their general practitioner.

Follow-up assessments took place every three months until month 18. At each visit, participants were asked about the occurrence of any UTIs, treatment adherence, and adverse events. Information on UTI episodes was confirmed where necessary from healthcare records. Blood samples were taken to monitor kidney and liver function in all participants. Urine samples were submitted to the central laboratory at baseline, at scheduled three monthly visits and at the time of UTI episodes. Optional perineal swabs were submitted at baseline and at six monthly routine visits. Participants completed symptom questionnaires every three months and at the time of symptomatic UTI.

### Outcomes

The primary clinical outcome measure was the incidence of symptomatic, antibiotic treated, UTI episodes self-reported by participants over the 12 month treatment period. An episode of UTI was defined as the presence of at least one symptom reported by patients or clinicians from a predefined list produced by Public Health England,^11^ together with the taking of a discrete treatment course of antibiotics for UTI. Consequently, the occurrence of the primary outcome was always defined by the independent prescribing clinician via confirmation of the likely diagnosis and recommendation of antibiotic treatment. The end of one UTI episode was defined as 14 days after the final antibiotic dose. If symptoms restarted or further antibiotics were prescribed within 14 days, this event was counted as the same episode.

Secondary outcomes were the incidence of symptomatic, antibiotic treated UTI in the six months after treatment; microbiologically confirmed UTIs; antibiotic resistance profiles in *Escherichia coli* isolated from urine and perineal swabs; asymptomatic bacteriuria; total antibiotic use; and hospital admissions due to UTI. Participant satisfaction with treatment was measured using the treatment satisfaction questionnaire for medication, which assessed four domains of treatment satisfaction: effectiveness, side effects, convenience, and global satisfaction.^12^

Microbiologically confirmed UTIs were episodes defined as per the primary outcome but with an additional criterion of positive urine culture at the time of UTI. A positive urine culture was defined as one isolate of a potential uropathogen at a concentration of ≥10^4^ colony forming units/mL or two species of uropathogens isolated at ≥10^5^ colony forming units/mL.^13^ Asymptomatic bacteriuria was defined as a positive urine culture from urine samples submitted to the central laboratory in the absence of symptoms. Antibiotic resistance was assessed from urine and perineal swabs with antimicrobial sensitivity tested in triplicate against a panel of antibiotic drugs. Multidrug resistance in *E coli* was defined as resistance to at least one antibiotic drug in at least three antimicrobial categories (supplementary material, page 1).^14^

### Statistical analysis

The trial was powered to assess non-inferiority of the absolute difference in UTI incidence over the 12 month treatment period. The non-inferiority margin, defined after a series of patient focus group meetings, was a difference of one UTI episode per year. Two meta-analyses of antibiotic prophylaxis and methenamine hippurate^8^ ^15^ quoted relative risks of UTI versus placebo of 0.15 and 0.24, respectively. With this information, and local audit suggesting that untreated participants had an average of 6.5 UTI episodes/year, we assumed an average incidence rate of 0.975 and 1.56 episodes/year in the antibiotic prophylaxis and methenamine hippurate groups, respectively, equating to an estimated difference of 0.6 episodes/year (in favour of antibiotics). Using a two sample t test, and assuming a difference of 0.6 episodes/year and a standard deviation of 0.9 (based on placebo groups from meta-analyses), we required 87 participants per group to be 90% sure that the lower limit of a one sided 95% confidence interval (equivalently 90% two sided) was above the non-inferiority limit. To account for an estimated 25% attrition rate over the course of the trial, the target sample size was 240.

For the primary outcome, the incidence rate in each group was the total number of UTI episodes divided by the total follow-up (exposure) time, reported with 95% confidence intervals calculated using a resampling procedure (bootstrap). The absolute difference between groups was calculated and reported with a 90% bootstrap confidence interval. Non-inferiority would be concluded if the upper limit of the confidence interval was below one UTI episode/year. We estimated relative treatment differences using a negative binomial model with follow-up time included as an exposure variable, recruiting centre as a random effect, and baseline stratification factors (menopausal status and prior UTI frequency) as fixed effects. This model yielded an estimate of the incidence rate ratio, presented with a 95% confidence interval. 

Sensitivity analyses excluded days when participants took therapeutic antibiotics for UTI from the follow-up time. We analysed a binary indicator of at least one UTI episode using a mixed effects logistic regression model adjusted for centre and baseline stratification factors. Analyses were primarily conducted in a modified intention-to-treat population, which included all participants observed for at least six months, because these participants were assumed to provide a reliable estimate of UTI incidence. Prespecified sensitivity analyses were conducted in intention-to-treat and per protocol populations (participants achieving ≥90% adherence with preventive treatment; switching between treatment strategies was considered adherent). We also conducted a post hoc sensitivity analysis in a strict per protocol population that included only those participants achieving ≥90% adherence with initially randomised treatment.

Symptomatically diagnosed UTI incidence in the post-treatment period and microbiologically confirmed UTIs were analysed as for the primary outcome measure. Secondary outcomes were analysed according to the intention-to-treat principle and included all participants with data available. Rates of asymptomatic bacteriuria and antibiotic resistance were compared by χ^2^ or Fisher’s exact tests. These analyses are considered exploratory and should be interpreted with caution. We made no adjustments for multiple testing. Domain scores of the treatment satisfaction questionnaire for drug treatment were compared between groups using a two sample t test and an analysis of covariance model adjusted for baseline stratification factors. To account for switching between treatment strategies, adverse events were reported according to treatment received at the time with data summarised by numbers of participants receiving each intervention. All analyses were conducted using Stata version 16. Independent trial steering and data monitoring committees had oversight throughout the trial. The trial was registered with the ISRCTN registry (ISRCTN70219762).

### Patient and public involvement

The host institution has an established UTI patient group and as such patient and public involvement began at the planning stage of this trial. Patient representatives were included on the trial steering committee. The patient and public involvement group helped define the main outcome measure and in particular stressed the importance of a practical UTI definition rather than sole reliance on microbiological tests. The non-inferiority margin was exclusively defined by the patient group who stressed the severity of UTI symptoms and advised a non-inferiority margin of one UTI in a year. This margin was considered a clinically meaningful difference by our patient and public involvement group based on an appreciation of the likely numerical reductions in UTI frequency in both of our trial arms. During recruitment, the study was advertised via patient representatives from Bladder Health UK and results will be disseminated widely among patients including via this groups’ regular publications.

## Results

### Participants

**Fig 1:**
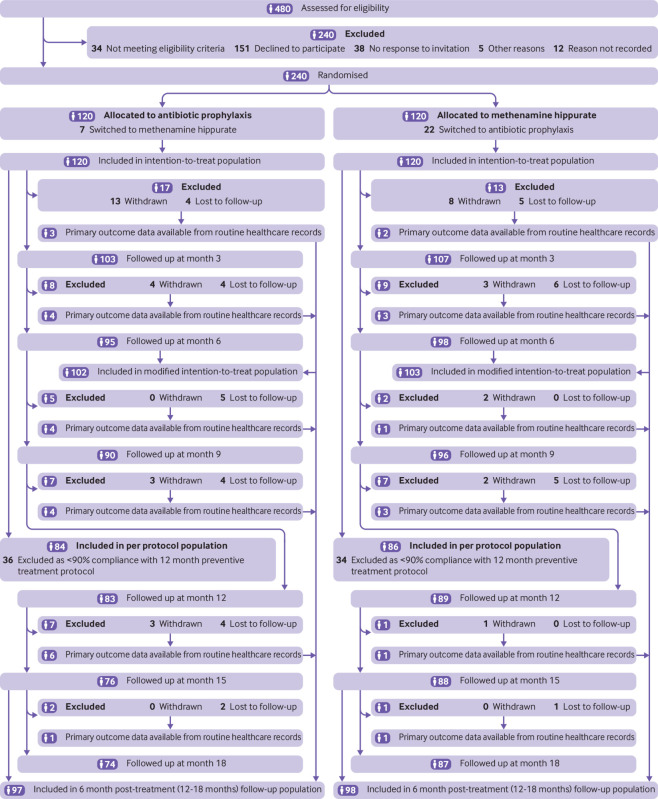
Trial profile (CONSORT flowchart)

Between 23 June 2016 and 20 June 2018, 240 participants were recruited and randomly assigned to antibiotic prophylaxis (n=120) or methenamine hippurate (n=120). For those allocated to antibiotic prophylaxis, 66 (55%) received nitrofurantoin, 30 (25%) trimethoprim, 24 (20%) cefalexin. A total of 22 (18%) participants allocated to methenamine hippurate switched to receive antibiotic prophylaxis and seven (6%) vice versa. Patient follow-up was completed in January 2020. The modified intention-to-treat analysis included 205 (85%) participants; 102 (85%) in the antibiotic arm and 103 (86%) in the methenamine hippurate arm (fig 1). Supplementary table S1 provides a summary of participants included in the primary and sensitivity analysis populations. Demographics and clinical characteristics at baseline were generally well balanced across treatment groups (table 1).

**Table 1:** Baseline characteristics. Data are number (%) of participants unless stated otherwise

	Intention-to-treat population			Modified intention-to-treat population	
	Antibiotic prophylaxis (n=120)	Methenamine hippurate (n=120)		Antibiotic prophylaxis (n=102)	Methenamine hippurate (n=103)
Mean (standard deviation) age (years)	50.3 (18.1)	49.9 (19.1)		51.1 (17.7)	51.1 (18.9)
Mean (standard deviation) weight (kg)	70.1 (15.3)	75.1 (18.5)		69.4 (14.6)	75.5 (18.5)
Menopausal status					
Pre-menopausal	49 (41)	50 (42)		40 (39)	41 (40)
Peri-menopausal/post-menopausal	71 (59)	70 (58)		62 (61)	62 (60)
No (%) of self-reported urinary tract infections in previous 12 months before trial entry					
<4	14 (12)	16 (13)		12 (12)	16 (16)
≥4	106 (88)	104 (87)		90 (88)	87 (84)
Median (interquartile range)	6 (4-8)	6 (4-8)		6 (4-8)	6 (4-8)
Mean (standard deviation)	6.8 (3.8)	7.0 (3.4)		6.6 (3.8)	6.7 (3.3)
No of positive urine culture reports in previous 12 months before trial entry*					
Median (interquartile range)	2 (1-4)	3 (1-5)		2 (1-4)	3 (1-5)
Previous use of antibiotic prophylaxis	28 (23)	27 (23)		23 (23)	22 (21)
Nitrofurantoin	20 (17)	20 (17)		17 (17)	17 (17)
Trimethoprim	16 (13)	11 (9)		13 (13)	9 (9)
Cefalexin	6 (5)	13 (11)		2 (2)	9 (9)
Co-amoxyclav	2 (2)	5 (4)		0 (0)	4 (4)
Amoxycillin	3 (3)	3 (3)		0 (0)	2 (2)
Ciprofloxacin	1 (1)	4 (3)		0 (0)	3 (3)
Pivmecillinam	1 (1)	3 (3)		1 (1)	3 (3)
Three month washout period required before randomisation	16 (13)	16 (13)		15 (15)	14 (14)
Previously taken methenamine hippurate	2 (2)	4 (3)		2 (2)	3 (3)
Results of central laboratory urine culture at baseline					
No growth	93 (78)	98 (82)		82 (80)	84 (82)
Growth of one or two isolates	18 (15)	13 (11)		16 (16)	13 (13)
No sample	9 (8)	9 (8)		4 (4)	6 (6)
Isolates identified from central laboratory urine culture at baseline					
* Escherichia coli*	15 (13)	7 (6)		14 (14)	7 (7)
Coliform—other	1 (1)	2 (2)		1 (1)	2 (2)
* Enterobacter cloacae* group	1 (1)	0		0	0
* Pseudomonas aeruginosa*	0	1 (1)		0	1 (1)
* Enterococcus faecalis*	1 (1)	0		1 (1)	0
* Staphylococcus aureus*	0	1 (1)		0	1 (1)
* Streptococcus agalactiae*	1 (1)	2 (2)		1 (1)	2 (2)
Resistance in any isolate identified from central laboratory urine culture at baseline					
Amoxicillin	9 (8)	6 (5)		8 (8)	6 (6)
Co-amoxiclav	3 (3)	1 (1)		2 (2)	1 (1)
Trimethoprim	9 (8)	3 (3)		8 (8)	3 (3)
Co-trimoxazole	3 (3)	1 (1)		2 (2)	1 (1)
Cefalexin	4 (3)	3 (3)		3 (3)	3 (3)
Cefuroxime	2 (2)	2 (2)		1 (1)	2 (2)
Cephalosporins—other	1 (1)	1 (1)		1 (1)	1 (1)
Ciprofloxacin	0	3 (3)		0	3 (3)
Gentamicin	1 (1)	1 (1)		1 (1)	1 (1)
Nitrofurantoin	1 (1)	1 (1)		0	1 (1)

* Data missing for three participants allocated to receive antibiotic prophylaxis and for three participants allocated to receive methenamine hippurate.

### Primary outcome

In the modified intention-to-treat population, 90 symptomatic, antibiotic treated UTI episodes were reported over 101 person years of follow-up in the antibiotic group, and 141 episodes over 102 person years of follow-up in the methenamine hippurate group (supplementary fig S1A). The incidence of symptomatic antibiotic treated UTI over the 12 month treatment period was therefore 0.89 episodes per person year (95% confidence interval 0.65 to 1.12) in the antibiotic group and 1.38 (1.05 to 1.72) in the methenamine hippurate group (absolute difference 0.49 (90% confidence interval 0.15 to 0.84)). With the upper limit of the 90% confidence interval below the non-inferiority limit of one, we can conclude methenamine hippurate to be non-inferior to antibiotic prophylaxis in this setting. This result was confirmed in all sensitivity analysis populations (table 2). To facilitate meta-analyses and comparisons with other studies, a 95% confidence interval for the primary outcome was estimated as 0.49 (0.08 to 0.90). On a relative scale, the adjusted incidence rate ratio was estimated as 1.52 (95% confidence interval 1.16 to 1.98) in favour of antibiotic prophylaxis. Secondary analysis of the primary outcome, excluding time spent taking therapeutic antibiotics for UTI from the follow-up time, showed consistent results (supplementary table S2). Supplementary table S3 shows the number and proportion of participants reporting at least one symptomatic, antibiotic treated UTI episode over the 12 month preventive treatment period.

**Table 2:** Incidence of episodes of symptomatic, antibiotic treated, urinary tract infection during 12 month preventive treatment period

Study population	No of participants included in analysis	Incidence rate (95% CI)	Absolute difference (90% CI)*	Incidence rate ratio (95% CI)†
**Modified intention to treat**‡				
Antibiotic prophylaxis	102	0.89 (0.65 to 1.12)	—	—
Methenamine hippurate	103	1.38 (1.05 to 1.72)	0.49 (0.15 to 0.84)§	1.52 (1.16 to 1.98)
**Strict intention to treat**¶				
Antibiotic prophylaxis	120	0.88 (0.65 to 1.11)	—	—
Methenamine hippurate	120	1.40 (1.08 to 1.73)	0.53 (0.20 to 0.86)	1.58 (1.24 to 2.03)
Per protocol**				
Antibiotic prophylaxis	84	0.87 (0.61 to 1.13)	—	—
Methenamine hippurate	86	1.29 (0.93 to 1.66)	0.42 (0.05 to 0.79)	1.44 (1.02 to 2.02)
**Post hoc, strict per protocol**††				
Antibiotic prophylaxis	82	0.83 (0.58 to 1.08)	—	—
Methenamine hippurate	71	1.13 (0.76 to 1.50)	0.30 (−0.08 to 0.67)	1.35 (1.06 to 1.71)

* Unadjusted absolute difference in incidence rate.

† Negative binomial model adjusted for menopausal status (pre-menopausal and peri-menopausal/post-menopausal), prior frequency of urinary tract infection (<4 and ≥4), and site (random effect).

‡ Modified intention to treat=primary analysis, including all patients with at least six months of follow-up data analysed according to their original treatment allocation.

§ Primary outcome.

¶ Strict intention to treat=including all patients who were randomised analysed according to their original treatment allocation.

** Per protocol=including all patients with at least six months of follow-up data who achieved ≥90% adherence with any trial preventive treatment analysed according to their original treatment allocation.

†† Post hoc, strict per protocol=including only those patients who achieved ≥90% adherence with their original allocated treatment, excluding those who changed treatment arm during the trial.

### Secondary outcomes

In the six month post treatment follow-up period, the UTI incidence rate was 1.19 (95% confidence interval 0.86 to 1.52) and 1.72 (1.27 to 2.18) episodes per person year in the antibiotic prophylaxis and methenamine hippurate groups, respectively (absolute difference 0.53 (95% confidence interval −0.03 to 1.09); supplementary table S4).

Overall, 183 (79%) of 231 UTI episodes reported in the modified intention-to-treat population were accompanied by a urine sample, and during the 12 month treatment period a positive urine culture was observed in 96 (52%) of these episodes. Incidence of microbiologically confirmed UTIs was 0.41 (95% confidence interval 0.27 to 0.56) in participants allocated to antibiotic prophylaxis and 0.53 (0.34 to 0.72) for those allocated methenamine hippurate (absolute difference 0.11 (−0.12 to 0.35); supplementary table S5). In the six months after treatment, 93 (66%) of 141 UTI episodes were associated with a urine sample, of which 65 (70%) were positive. Incidence of microbiologically confirmed UTI episodes was 0.48 (0.28 to 0.68) and 0.86 (0.59 to 1.14) in the antibiotic prophylaxis and methenamine hippurate groups, respectively (absolute difference 0.38 (0.04 to 0.72)).

During the 18 month trial period, the rate of asymptomatic bacteriuria was similar between treatment groups, however, a post hoc analysis of urine samples submitted during the 12 month treatment period identified a significantly higher rate in the methenamine hippurate group than in the antibiotic prophylaxis group (44 (14%) of 326 samples *v* 22 (7%) of 323; χ^2^ test, P=0.0048; supplementary table S6).

Therapeutic antibiotics for UTI were received during the 12 month treatment period by 51 (43%) and 67 (56%) participants allocated to the antibiotic prophylaxis and methenamine hippurate groups, respectively. In the six month observation period, the total number of days spent taking therapeutic antibiotics was higher in the methenamine hippurate group than in the antibiotic prophylaxis group (13.5 (interquartile range 6.5-23) *v* 7.5 (4-15)). Similarly, antibiotics for other infections were received by 28 (29%) of 98 participants receiving methenamine hippurate compared with 15 (15%) of 97 receiving antibiotic prophylaxis (supplementary table S7).

### Antimicrobial resistance

**Fig 2:**
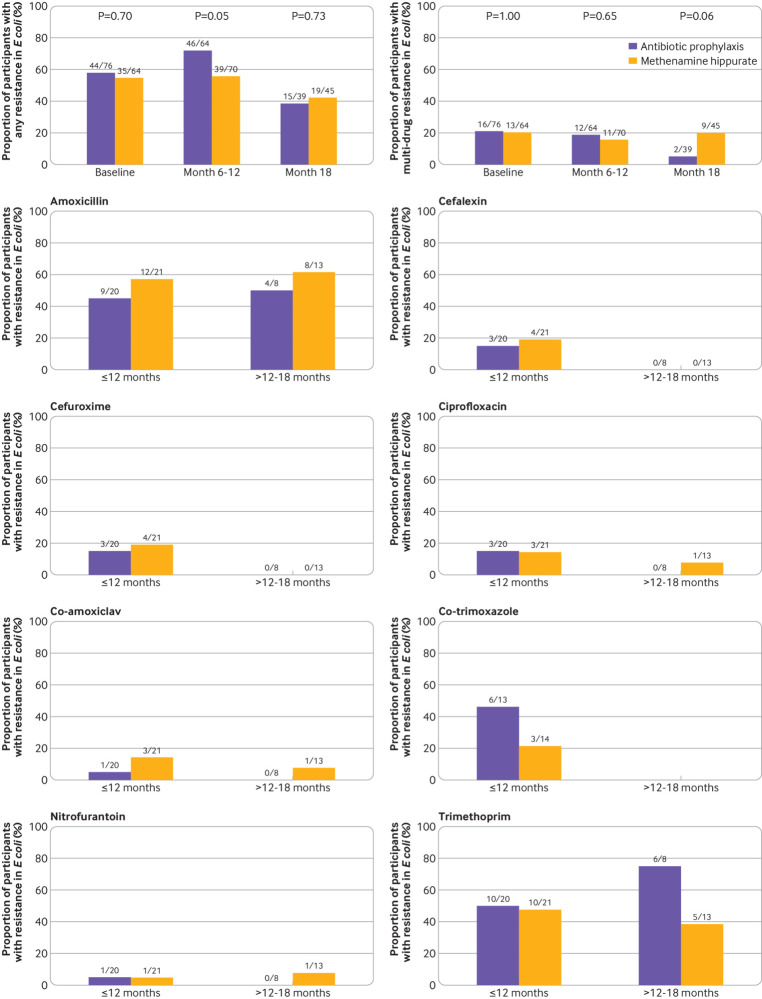
Antibiotic resistance rates in *Escherichia coli* isolated from perineal swabs, and urine samples taken during an episode of symptomatic urinary tract infection. Top left panel: proportion of participants (with *E coli* isolated) showing resistance to at least one antibiotic in *E coli* isolated from perineal swabs at baseline, six or 12 month follow-up, and 18 month follow-up (P values from χ^2^ test). Top right panel: proportion of participants (with *E coli* isolated) showing multidrug resistance in *E coli* isolated from perineal swabs at baseline, six or 12 month follow-up, and 18 month follow-up (P values from Fisher’s exact test). Remaining rows: proportion of participants showing resistance in *E coli* isolated from any symptomatic urine sample submitted during the 12 month preventive treatment period or six month observational period after treatment (out of those participants with *E coli* isolated from a symptomatic urine sample)

Availability of optional six monthly perineal swabs over the 18 month trial period is presented in supplementary table S8. The proportion of participants demonstrating resistance to at least one antibiotic in *E coli* isolated from perineal swabs was similar between randomised groups at baseline. At six or 12 month follow-up, this proportion was higher in the antibiotic prophylaxis group than in the methenamine hippurate group (46/64 (72%) *v* 39/70 (56%); χ^2^ test, P=0.05, fig 2, top left panel). However, at month 18, multidrug resistance in *E coli* isolated from perineal swabs was higher in the methenamine hippurate group than in the antibiotic prophylaxis group (9/45 (20%) *v* 2/39 (5%), Fisher’s exact test, P=0.06, fig 2, top right panel).

A substantial growth of *E coli* was isolated from the urine samples of 41 participants during 67 episodes of symptomatic UTI (that is, symptomatic urine samples) over the 12 month treatment period, and from 21 participants during 26 symptomatic episodes over the six months after treatment. The proportion of participants showing resistance to at least one of the antimicrobial drugs tested in *E coli* isolated from symptomatic urine samples was similar between randomised treatment groups (supplementary fig S2A), as were the rates of multidrug resistance (supplementary fig S2B). However, a higher proportion of participants in the antibiotic prophylaxis group than in the methenamine hippurate showed resistance to co-trimoxazole (6/13 (46%) *v* 3/14 (21%)) during the 12 month treatment period, and resistance to trimethoprim (6/8 (75%) *v* 5/13 (38%)) during the six months after treatment (fig 2). Similarly, a higher proportion of *E coli* isolates from symptomatic urine samples submitted during the 12 month treatment period demonstrated resistance to cephalosporins in those allocated to antibiotic prophylaxis compared to methenamine hippurate (supplementary fig S3). In contrast to the numbers of positive samples collected during symptomatic episodes, *E coli* was isolated from only a small proportion of urine samples collected routinely at three month intervals from asymptomatic participants (supplementary table S9A). All isolates identified from urine samples are listed in supplementary table S9B.

### Treatment satisfaction

On average, treatment satisfaction was high and generally comparable between treatment groups, although the antibiotic prophylaxis group reported higher scores in the convenience domain than the methenamine hippurate group (mean 91.4 (standard deviation 12.7) *v* 82.2 (18.4); t test, P=0.001; supplementary table S10).

### Adverse events

Rates of adverse events and adverse reactions were low and comparable across treatment groups (table 3). Two serious adverse reactions (severe abdominal pain and raised alanine transaminase) were reported, both in participants allocated to antibiotic prophylaxis. Kidney and liver function was assessed by blood tests taken every three months. We saw little difference in the distribution of these measurements between treatment groups or over time (supplementary fig S4). Over the 18 month trial period, four participants allocated to methenamine hippurate were admitted to hospital because of UTI. Six participants who were allocated to methenamine hippurate reported a fever of ≥38°C during a UTI episode (febrile UTI).

**Table 3:** Adverse events. Data are number (%) of participants or mean (standard deviation)

	Antibiotic prophylaxis (n=142*)	Methenamine hippurate (n=127*)
No of adverse events reported per participant	1.9 (2.8)	1.8 (2.4)
Worst grade adverse event reported per participant		
None	59 (42)	45 (35)
Mild	41 (29)	47 (37)
Moderate	34 (24)	29 (23)
Severe	8 (6)	6 (5)
No of adverse reactions reported per participant	0.4 (0.7)	0.4 (0.7)
No of participants reporting an adverse reaction	34 (24)	35 (28)
Worst grade adverse reaction reported per participant		
None	108 (76)	92 (72)
Mild	24 (17)	26 (20)
Moderate	9 (6)	9 (7)
Severe	1 (1)	0
No of participants affected by each adverse event†		
Lower respiratory tract infection	10 (7)	9 (7)
Nausea	12 (8)	5 (4)
Abdominal pain	7 (5)	9 (7)
Diarrhoea	8 (6)	4 (3)
Alanine aminotransferase increased	5 (4)	5 (4)
Back pain	7 (5)	3 (2)
Headache	3 (2)	7 (6)
Candida infection	4 (3)	5 (4)
Dyspepsia	5 (4)	4 (3)
Rash	3 (2)	5 (4)
Abdominal discomfort	3 (2)	4 (3)
Dyspnoea	5 (4)	2 (2)
Fall	3 (2)	4 (3)
Vomiting	3 (2)	4 (3)
Depressed mood	1 (1)	4 (3)
Herpes zoster	5 (4)	0

* Numbers of participants receiving each treatment.

† Only those events occurring in at least 3% of participants in either group are reported.

## Discussion

This trial has demonstrated that the non-antibiotic preventive treatment for UTI (methenamine hippurate) is not inferior to the current guideline recommended standard (daily, low dose prophylactic antibiotics). This trial adds to the evidence base for the use of methenamine hippurate for prophylactic treatment in adult women with recurrent UTI. Although the methenamine hippurate group had a 55% higher rate of UTI episodes than the antibiotics group, the absolute difference was just 0.49 UTI episodes per year, which has limited clinical consequence.

The risks of long term prophylactic antibiotic treatment have been well recognised in a recent study,^16^ which identified the increased risk of antibiotic resistance development in elderly patients receiving this treatment. The authors also described an increased risk of antibiotic associated complications, including *Clostridium difficile* infection, and concluded that the risks of long term antibiotic prophylaxis might outweigh the benefits in older patients with UTI. Our results could support a change in practice in terms of preventive treatments for recurrent UTI and provide patients and clinicians with a credible alternative to daily antibiotics, giving them the confidence to pursue strategies that avoid long term antibiotic use. The observed numerical difference in clinically diagnosed UTI between the two trial arms, which favoured antibiotic prophylaxis, was small and did not exceed the predefined non-inferiority margin of one episode per person year. This finding was consistent across the modified intention-to-treat, strict intention-to-treat, per protocol, and modified per protocol (post hoc) analyses. The information provided by this trial will allow clinicians and patients to undertake a shared decision making process relating to UTI preventive treatments. The study showed a small numerical difference in UTI incidence between the daily antibiotics and methenamine hippurate groups, but the potential trade-off includes the avoidance of antibiotic consumption, which is closely associated with antimicrobial resistance development.

Reductions in clinically diagnosed UTI during the treatment period were in line with previously published results from systematic reviews^8^ ^15^ and similar in both arms, confirming efficacy for both treatments. Around half of all participants were UTI-free during the treatment period (UTI-free rate=43% for the methenamine hippurate group, 54% for the antibiotics group).

Microbiological testing of urine samples during the treatment period showed that the use of positive urine culture as a criterion for UTI diagnosis would have resulted in a failure to capture around half (48%) of all patient reported episodes treated with antibiotics. This proportion is in line with results from another trial that reported only 58% of clinical UTIs confirmed by a positive urine culture.^17^ The discordance between clinical and microbiological UTI definitions suggests that a clinical definition of UTI is better aligned with actual clinical practice given that most urinary tract infections are treated before knowledge of microbiological culture results. Furthermore, some guidelines now encourage symptom based diagnosis of UTI and recommend a move away from reliance on microbiological culture.^18^ The latest Scottish Intercollegiate Guidelines Network publication underlines this, stating that “Routine urine culture is not required to manage LUTI [lower urinary tract infection] in women” and “Women with symptomatic LUTI should receive empirical antibiotic treatment.”^18^

The two trial treatments are licensed for UTI prevention^9^; therefore, as expected, the adverse event rate was low. Only two serious adverse events (abdominal pain requiring hospital admission and severe derangement in liver function tests) were classified as possibly or probably related to trial drug treatment and both occurred in the antibiotic arm. The rates of hospital admission for UTI (four participants) and febrile UTI (≥38°C; six participants) were low, but these participants were all in the methenamine hippurate arm. This information regarding an increased incidence of severe UTIs in the methenamine hippurate group can also be used by patients and clinicians in the decision making process when choosing UTI preventive treatments.

During the six month follow-up period after treatment, UTI rates increased but remained substantially lower than rates before treatment. These findings contrast those from a Cochrane review of antibiotic prophylaxis,^15^ which suggested that UTI rates were high after a long course of low dose antibiotics. The 12 month treatment period in this trial might explain this finding because most trials included in previous systematic reviews analysed antibiotic use for six months only.

In this trial, the use of methenamine hippurate as a preventive treatment against recurrent UTI was associated with a reduction in overall antibiotic use and equivalent levels of treatment satisfaction compared to daily antibiotics. Treatment satisfaction domains explored were effectiveness, side effects, convenience, and global satisfaction, and we saw no differences between arms in individual domain scores apart from convenience. Participants scored once daily antibiotics as more convenient than twice daily methenamine hippurate (supplementary table S10). In the methenamine hippurate arm, 44% of women did not receive any therapeutic antibiotics during the 12 month treatment period, which is directly aligned to current antibiotic stewardship strategies designed to reduce antimicrobial resistance.^7^

Antimicrobial resistance development associated with both trial treatments was explored in cultures from both perineal swabs and urine samples collected throughout the trial. Overall rates of resistance to nitrofurantoin were very low (fig 2). During the treatment period, a higher proportion of patients allocated to daily prophylactic antibiotics showed resistance to at least one antibiotic in *E coli* isolates from perineal swabs than patients allocated to methenamine hippurate. Results from urine cultures revealed higher rates of resistance to trimethoprim, co-trimoxazole, and cephalosporins in *E coli* isolated from urine samples from women in the antibiotic arm than in the methenamine hippurate arm. The described differences in antimicrobial resistance rates suggest that the use of continuous, low dose, antibiotic prophylaxis is a contributory factor to the development of antimicrobial resistance and is similar to findings from a previous randomised controlled trial exploring antibiotic prophylaxis in patients with recurrent complicated UTIs.^17^ By contrast, at the end of the follow-up period, the rate of multidrug resistance in *E coli* isolates from perineal swabs was higher in the methenamine hippurate arm than in the antibiotics arm. This difference could be due to a sustained effect of daily antibiotics on the faecal microbiome or the greater incidence of antibiotic treated acute UTIs in the methenamine hippurate group during follow-up.

This trial was conducted in line with current best practice and all outcomes were defined a priori and reflected those important to both patients and clinicians. Primary outcome allocation was verified from healthcare records and a random sample group of participants was examined by an independent clinician, blinded to treatment allocation. This independent verification agreed with the allocation of primary outcome in all cases.

The adherence of participants to their allocated treatment was regularly assessed, and results showed that the vast majority of participants were over 90% adherent with the allocated treatment. A realistic attrition rate, given the 18 month trial period, was incorporated into the sample size calculation and the number of withdrawals was in line with predicted rates. All pre-set thresholds regarding numbers of participants contributing to the primary analysis were met.

The trial was designed in a pragmatic fashion to allow for widespread applicability and generalisability. A broad range of eligible participants accurately represented women with recurrent UTI seen regularly in routine NHS practice. Women from a range of geographical and socioeconomic areas were included in the trial and shared decision making between patients and clinicians regarding choice of antibiotic was in line with good clinical practice. Patients were allowed to crossover between trial arms, which again reflects usual care.

### Limitations of this study

Limitations of the ALTAR trial included the lack of blinding, which would have increased the certainty of results but would have hugely increased trial costs. The treatment of any breakthrough UTIs was decided by healthcare providers who were not involved in the study and had no influence on trial results. Another limitation was the heterogeneity of prophylactic antibiotics prescribed, which alongside the wide inclusion criteria prevented meaningful subgroup analysis that could identify differences in efficacy of individual antibiotic drugs or specific subgroups of patients who might gain particular benefit from either of the trial treatments. Further research should focus on the use of methenamine hippurate as a preventive treatment for recurrent UTI in more narrowly defined patient groups. In addition, the added value of urinary acidification was not explored in this study despite the practice of some clinicians who prescribe vitamin C alongside methenamine hippurate to encourage acidic urine. Finally, data regarding long term safety of methenamine hippurate are scarce, and this question was outside of the scope of the current trial. Increased adoption of this treatment as prophylaxis against recurrent UTI will allow for the generation of long term safety data now that efficacy has been demonstrated in our study.

### Conclusions

In the ALTAR trial, we have demonstrated high levels of efficacy from methenamine hippurate in terms of UTI prevention, and have shown that this efficacy is comparable to the current guideline recommended prophylaxis (that is, long course, low dose antibiotic treatment). The increased rates of antimicrobial resistance development associated with the antibiotic arm as shown in the primary uropathogen *E coli* might encourage patients and clinicians to consider methenamine hippurate as a first line treatment for UTI prevention in women.

**What is already known on this topic**

Long term, low dose daily antibiotic treatment is the current standard of care for prophylaxis in women with recurrent urinary tract infection and is recommended by national and international guidelines
Despite the reported success of prophylactic antibiotics, antimicrobial resistance has been directly linked to antibiotic consumption; as a result, the importance of research into non-antibiotic alternatives has been highlighted
Systematic reviews have concluded that the non-antibiotic option of methenamine hippurate could be effective in preventing urinary tract infections, but outlined the need for further large, well conducted randomised trials

**What this study adds**

The ALTAR randomised trial compared use of methenamine hippurate with low dose antibiotics in a predefined cohort of women with recurrent urinary tract infection presenting to secondary care
Efficacy of both treatments in the primary and sensitivity analyses was found to be comparable, suggesting that methenamine hippurate might be appropriate for women with a history of recurrent urinary tract infection
The range of a priori outcomes reported confirm clinical utility of methenamine hippurate as a non-antibiotic option for prevention of urinary tract infection in this pragmatic trial, which allows generalisability of results

## Data Availability

All data requests should be submitted to the corresponding author for consideration. Access to de-identified data collected during the trial, alongside a data dictionary, may be granted to researchers who submit a methodologically sound proposal. To gain access, data requestors will need to complete forms required as part of the application process.

## Web extra

Extra material supplied by authors

Web appendix: Supplementary information

## Notes

Contributors: CH, TC, LV, JL, RP, IE, IP, NT, KG, KW, and AAl designed the trial and provided intellectual input into the study protocol. CH, IE, IP, NT, KG, ZH, HL, and AAl recruited participants and collected data. HM conducted the main study analysis, under the supervision of TC. KW led the microbiological aspects of the study. GW, RF, and SCu set up and managed the study under the supervision of SCa. AAb designed and set up databases and managed central data processes. CH and HM cowrote the manuscript. CH was the chief investigator and guarantor. RP is deceased. All authors reviewed and commented on the manuscript before submission and gave approval to submit for publication. The corresponding author had full access to all the data in the study and had final responsibility for the decision to submit for publication. The corresponding author attests that all listed authors meet authorship criteria and that no others meeting the criteria have been omitted.

The lead author affirms that the manuscript is an honest, accurate, and transparent account of the study being reported; that no important aspects of the study have been omitted; and that any discrepancies from the study as planned (and, if relevant, registered) have been explained.

Dissemination to participants and related patient and public communities: All participants will be sent a summary of trial results and further dissemination will be via a variety of sources. The patient representative on our trial steering committee is a member of the patient support group, Bladder Health UK, and has been asked to write a summary of trial results for their quarterly publication. Finally, we have had a great deal of interest regarding the trial outcome from worldwide patient groups (via social media) and we aim to disseminate our findings using Twitter.

Provenance and peer review: Not commissioned; externally peer reviewed.
