# Supplementary material for: Alternative to prophylactic antibiotics for the treatment of recurrent urinary tract infections in women: multicentre, open label, randomised, non-inferiority trial
Source: BMJ. 2022 Mar 9;376:e068229. doi: 10.1136/bmj-2021-0068229 (PMC8905684; doi:10.1136/bmj-2021-0068229)
Supplement: Supplementary file 1 — Web appendix: Supplementary information [file harc068229.ww.pdf]

SUPPLEMENTARY INFORMATION

Attribution of the primary outcome measure

The primary outcome measure was collected across multiple data sources; participant-completed UTI logs, 3-monthly participant-reported questionnaires, and 3-monthly site-reported case report forms. Episodes of UTI were identified by the trial statistician using statistical programming, using the following pre-defined hierarchy of evidence to avoid double counting:

1. Data from healthcare records
2. Participant-reported UTI log completed at the time of UTI
3. Phone-reported UTI log completed at the time of UTI
4. 3-monthly site-reported case report forms
5. 3-monthly participant-reported questionnaires

An independent clinician not otherwise involved in the trial reviewed primary outcome data, blind to treatment allocation, for a 10% sample of cases and was asked to identify symptomatic UTI episodes following the same hierarchy. The number of UTI episodes was found to match in all cases when following the pre-defined criteria.

Multi-drug resistance definition

Multi-drug resistance was defined as resistance in *E. coli* to at least one antimicrobial agent in at least three antimicrobial categories, following the principles described by Magiorakos *et al.* The antimicrobial agents and categories were tailored to be specific to uropathogens and are given below:

**Antimicrobial category**	**Antimicrobial agent**
Aminoglycosides	gentamicin
Antipseudomonal penicillin	piperacillin/tazobactam
Carbapenems	ertapenem; meropenem
Non-extended spectrum cephalosporins	cefuroxime; cefalexin
Fluoroquinolones	ciprofloxacin
Folate pathway inhibitors	trimethoprim-sulphamethoxazole(co-trimoxazole); trimethoprim
Monobactams	aztreonam
Penicillins	amoxicillin; mecillinam;
Penicillins + β-lactamase inhibitors	amoxicillin-clavulanic acid (co-amoxiclav)
β-lactamase resistant penicillin	temocillin
Phosphonic acids	fosfomycin
Nitrofuran	nitrofurantoin

Magiorakos AP, Srinivasan A, Carey RB, Carmeli Y, Falagas ME, Giske CG, *et al.* Multidrug-resistant, extensively drug-resistant and pandrug-resistant bacteria: an international expert proposal for interim standard definitions for acquired resistance. *Clin Microbiol Infect* 2012;**18**:268-81. https://doi.org/10.1111/j.1469-0691.2011.03570.x

Table s1: Summary of analysis populations

	12-month treatment period								6-month post-treatment observational period	
	Modified ITT		ITT		Per-protocol		Strict per-protocol*			
	Antibiotic prophylaxis	Methenamine hippurate	Antibiotic prophylaxis	Methenamine hippurate	Antibiotic prophylaxis	Methenamine hippurate	Antibiotic prophylaxis	Methenamine hippurate	Antibiotic prophylaxis	Methenamine hippurate
Number analysed	102 (85%)	103 (86%)	120 (100%)	120 (100%)	84 (70%)	86 (72%)	82 (68%)	71 (59%)	97 (81%)	98 (82%)
≥90% compliance with any trial preventative treatment	84 (82%)	86 (83%)	84 (70%)	86 (72%)	84 (100%)	86 (100%)	82 (100%)	71 (100%)	82 (85%)	85 (87%)
Received alternative treatment strategy	7 (7%)	19 (18%)	7 (6%)	22 (18%)	2 (2%)	15 (17%)	0 (0%)	0 (0%)	5 (5%)	15 (15%)
Follow-up time (days)										
Mean (SD)	362.6 (14.5)	361.6 (18.0)	319.4 (105.6)	323.1 (97.8)	364.7 (2.9)	364.8 (1.7)	365.0 (0.0)	365.0 (0.0)	179.9 (11.2)	181.4 (7.0)
Min, Max	239, 365	218, 365	14, 365	35, 365	338, 365	349, 365	365, 365	365, 365	83, 183	120, 183
Menopausal status										
Pre	40 (39%)	41 (40%)	49 (41%)	50 (42%)	30 (36%)	34 (40%)	29 (35%)	32 (45%)	35 (36%)	39 (40%)
Peri/Post	62 (61%)	62 (60%)	71 (59%)	70 (58%)	54 (64%)	52 (60%)	53 (65%)	39 (55%)	62 (64%)	59 (60%)
Self-reported urinary tract infections in the 12 months before trial entry										
<4	12 (12%)	16 (16%)	14 (12%)	16 (13%)	11 (13%)	14 (16%)	11 (13%)	12 (17%)	12 (12%)	16 (16%)
≥4	90 (88%)	87 (84%)	106 (88%)	104 (87%)	73 (87%)	72 (84%)	71 (87%)	59 (83%)	85 (88%)	82 (84%)

*Post-hoc analysis. ITT: intention-to-treat

- Modified intention to treat - this was the primary analysis and included all patients with at least 6 months of follow-up data analysed according to their original treatment allocation.
- Strict intention to treat - this included all patients who were randomised analysed according to their original treatment allocation.
- Per protocol – this included all patients with at least 6 months of follow-up data who achieved 90% or greater compliance with any trial preventative treatment analysed according to their original treatment allocation.
- Post-hoc strict per protocol - this included only those patients who achieved 90% or greater compliance with their original allocated treatment, excluding those who changed treatment arm during the trial.

Figure s1: Frequency of symptomatic, antibiotic-treated UTI episodes

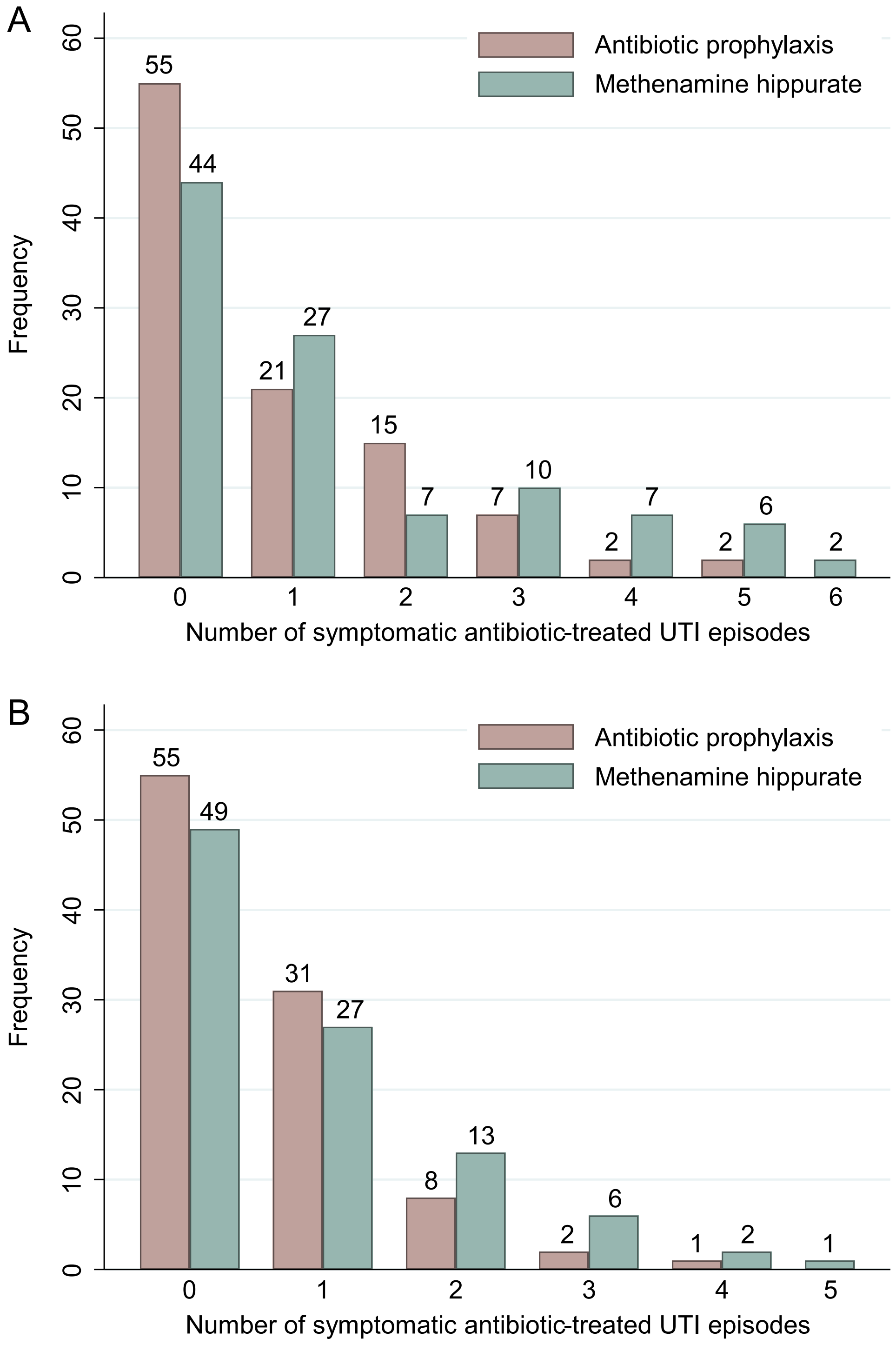

(A) Frequency of symptomatic antibiotic-treated UTI episodes during the 12-month preventative treatment period (modified intention-to-treat population). **(B)** Frequency of symptomatic antibiotic-treated UTI episodes during the 6-month post-treatment observational period.

Table s2: Secondary analysis of the primary outcome, excluding time taking therapeutic antibiotics for UTI from follow-up (exposure) time

	Number included in analysis	Incidence rate (95% CI)	Absolute difference (90% CI)	Incidence rate ratio‡ (95% CI)
Modified intention-to-treat				
Antibiotic prophylaxis	102	0·91 (0·66-1·15)	··	··
Methenamine hippurate	103	1·43 (1·07-1·79)	0·52 (0·16-0·89)	1·55 (1·17-2·06)
Intention-to-treat				
Antibiotic prophylaxis	120	0·89 (0·65-1·13)	··	··
Methenamine hippurate	120	1·45 (1·11-1·80)	0·56 (0·21-0·91)	1·62 (1·26-2·09)
Per-protocol				
Antibiotic prophylaxis	84	0·89 (0·61-1·16)	··	··
Methenamine hippurate	86	1·34 (0·95-1·72)	0·45 (0·05-0·84)	1·48 (1·03-2·13)
Strict per-protocol				
Antibiotic prophylaxis	82	0·84 (0·59-1·10)	··	··
Methenamine hippurate	71	1·16 (0·77-1·55)	0·32 (-0·08-0·71)	1·38 (1·07-1·79)

‡ Negative binomial model adjusted for menopausal status (pre and peri/post), prior UTI frequency (<4 and ≥4) and site (random effect).

Table s3: Number of participants reporting at least one episode of symptomatic, antibiotic-treated UTI

	Antibiotic prophylaxis	Methenamine hippurate	Odds Ratio (95% CI)‡
**12-month treatment period**			
Modified intention-to-treat	47/102 (46%)	59/103 (57%)	1·55 (0·88-2·74)
Intention-to-treat	49/120 (41%)	64/120 (53%)	1·69 (1·00-2·87)
Per-protocol	39/84 (46%)	45/86 (52%)	1·29 (0·69-2·41)
Strict per-protocol	38/82 (46%)	35/71 (49%)	1·14 (0·59-2·21)
**6-month post-treatment observation period**	42/97 (43%)	49/98 (50%)	1·32 (0·73-2·39)

Data are n/N (%) unless otherwise stated. ‡ Logistic regression model adjusted for menopausal status (pre and peri/post), prior UTI frequency (<4 and ≥4) and site (random effect).

Table s4: Incidence of symptomatic, antibiotic-treated UTI episodes during the 6-month post-treatment observation period

	Number included in analysis	Incidence rate (95% CI)	Absolute difference (95% CI)	Incidence rate ratio‡ (95% CI)
Antibiotic prophylaxis	97	1·19 (0·86-1·52)	··	··
Methenamine hippurate	98	1·72 (1·27-2·18)	0·53 (-0·03-1·09)	1·45 (1·16-1·81)

‡ Negative binomial model adjusted for menopausal status (pre and peri/post), prior UTI frequency (<4 and ≥4) and site (random effect).

Table s5: Episodes of microbiologically-confirmed symptomatic, antibiotic-treated UTI

	Number included in analysis	Incidence rate (95% CI)	Absolute difference (95% CI)	Incidence rate ratio (95% CI)
**12-month treatment period**				
Modified intention-to-treat				
Antibiotic prophylaxis	102	0·41 (0·27-0·56)	··	··
Methenamine hippurate	103	0·53 (0·34-0·72)	0·11 (-0·12-0·35)	1·25 (1·05-1·49) ‡
**6-month post-treatment observation period**				
Antibiotic prophylaxis	97	0·48 (0·28-0·68)	··	··
Methenamine hippurate	98	0·86 (0·59-1·14)	0·38 (0·04-0·72)	1·86 (1·27-2·73)†

‡ Negative binomial model adjusted for menopausal status (pre and peri/post), prior UTI frequency (<4 and ≥4) and site (random effect). † Poisson model adjusted for menopausal status (pre and peri/post), prior UTI frequency (<4 and ≥4) and site (random effect). A Poisson model was as this proved a better model fit than the negative binomial model.

Table s6: Asymptomatic bacteriuria

	Antibiotic prophylaxis (N=120)	Methenamine hippurate (N=120)	χ^2 p-value
Baseline	18/111 (16%)	13/111 (12%)	-
During treatment	22/323 (7%)	44/326 (14%)	0·0048
During follow-up	24/123 (20%)	22/134 (16%)	0·52

Data are number of positive urine cultures / number of routine urine samples (%)

Table s7: Therapeutic antibiotic use during the 12-month treatment period and 6-month post-treatment observation period

	12-month treatment period		6-month post-treatment period	
	Antibiotic Prophylaxis (N = 120)	Methenamine Hippurate (N = 120)	Antibiotic Prophylaxis (N = 97)	Methenamine Hippurate (N = 98)
**Therapeutic antibiotics for UTI**				
No. receiving ≥1 day of treatment	51 (43%)	67 (56%)	48 (49%)	52 (53%)
Antibiotic(s) received*				
Nitrofurantoin	33 (65%)	47 (70%)	33 (69%)	42 (81%)
Trimethoprim	21 (41%)	17 (25%)	13 (27%)	9 (17%)
Cefalexin	8 (16%)	11 (16%)	3 (6%)	8 (15%)
Meropenem	0 (0%)	0 (0%)	0 (0%)	1 (2%)
Cefuroxime	0 (0%)	1 (1%)	0 (0%)	0 (0%)
Penicillin V	0 (0%)	1 (1%)	0 (0%)	0 (0%)
Ampicillin	0 (0%)	1 (1%)	0 (0%)	0 (0%)
Amoxicillin	6 (12%)	8 (12%)	5 (10%)	4 (8%)
Co-amoxiclav	7 (14%)	6 (9%)	1 (2%)	0 (0%)
Doxycyclin	1 (2%)	0 (0%)	0 (0%)	0 (0%)
Gentamicin	0 (0%)	2 (3%)	0 (0%)	0 (0%)
Clarithromycin	1 (2%)	1 (1%)	0 (0%)	0 (0%)
Metronidazole	0 (0%)	2 (3%)	0 (0%)	1 (2%)
Ciprofloxacin	7 (14%)	9 (13%)	3 (6%)	5 (10%)
Pivmecillinam	7 (14%)	9 (13%)	5 (10%)	6 (12%)
Fosfomycin	0 (0%)	0 (0%)	1 (2%)	0 (0%)
Total days of treatment	13 (8-19)	16 (7-25)	7·5 (4-15)	13·5 (6·5-23)
**Therapeutic antibiotics for other infections**				
No. receiving ≥1 day of treatment	32 (27%)	38 (32%)	15 (15%)	28 (29%)
Total days of treatment	10·5 (7-20.5)	9 (6-19)	8 (7-12)	8 (5·5-13)

*Number of participants receiving each antibiotic. % of those receiving ≥1 day of therapeutic antibiotic treatment for UTI.

Data are n(%), or median (IQR)

Table s8: Antibiotic resistance in *E. coli* isolated from perineal swabs

	Antibiotic prophylaxis (N=120)				Methenamine hippurate (N=120)			
	Baseline	Month 6	Month 12	Month 18	Baseline	Month 6	Month 12	Month 18
Samples available	107 (89%)	75 (63%)	66 (55%)	59 (49%)	94 (78%)	79 (66%)	70 (58%)	62 (52%)
E. coli isolated	76 (71%)	51 (68%)	43 (65%)	39 (66%)	64 (68%)	58 (73%)	47 (67%)	45 (73%)
Any antibiotic resistance in *E. coli*	44 (58%)	32 (63%)	30 (70%)	15 (38%)	35 (55%)	31 (53%)	24 (51%)	19 (42%)
Resistance in *E. coli* (number of antibiotics)	1 (0-3); 0-8	1 (0-2); 0-6	1 (0-2); 0-7	0 (0-2); 0-4	1 (0-3); 0-9	1 (0-2); 0-6	1 (0-2); 0-6	0 (0-2); 0-8
Multi-drug resistance in *E. coli*	16 (21%)	8 (16%)	8 (19%)	2 (5%)	13 (20%)	7 (12%)	6 (13%)	9 (20%)

Data are n (%) or median (IQR); range.

Table s9a: Availability of 3-monthly urine samples

	Antibiotic prophylaxis (N=120)		Methenamine hippurate (N=120)	
	Samples available	*E. coli* isolated	Samples available	*E. coli* isolated
Baseline	111 (93%)	15 (14%)	111 (93%)	7 (6%)
Month 3	95 (79%)	2 (2%)	96 (80%)	8 (8%)
Month 6	81 (68%)	4 (5%)	89 (74%)	9 (10%)
Month 9	79 (66%)	3 (4%)	81 (68%)	5 (6%)
Month 12	74 (62%)	5 (7%)	78 (65%)	12 (15%)
Month 15	64 (53%)	11 (17%)	71 (59%)	8 (11%)
Month 18	62 (52%)	7 (11%)	71 (59%)	8 (11%)

Table s9b: Isolates identified from all urine samples

Isolate	Antibiotic prophylaxis	Methenamine hippurate
Escherichia coli	84 (71%)	114 (66%)
Coliform Other	6 (5%)	15 (9%)
Enterococcus faecalis	10 (8%)	9 (5%)
Klebsiella pneumonia	2 (2%)	13 (8%)
Pseudomonas aeruginosa	1 (1%)	8 (5%)
Streptococcus agalactiae	3 (3%)	4 (2%)
Acinetobacter spr	4 (3%)	1 (1%)
Staphylococcus aureus	0 (0%)	3 (2%)
Proteus sp	1 (1%)	1 (1%)
Proteus mirabilis	2 (2%)	0 (0%)
Klebsiella oxytoca	1 (1%)	1 (1%)
Enterobacter cloacae group	1 (1%)	0 (0%)
Citrobacter freundii group	0 (0%)	1 (1%)
Pseudomonas sp	0 (0%)	1 (1%)
Enterococcus faecium	1 (1%)	0 (0%)
Streptococcus sp	1 (1%)	0 (0%)
Streptococcus bovis	0 (0%)	1 (1%)
Candida albicans	1 (1%)	0 (0%)
Total	118	172

Figure s2: Antibiotic resistance in symptomatic urine samples submitted during the 12-month preventive treatment period and 6-month post-treatment observational period

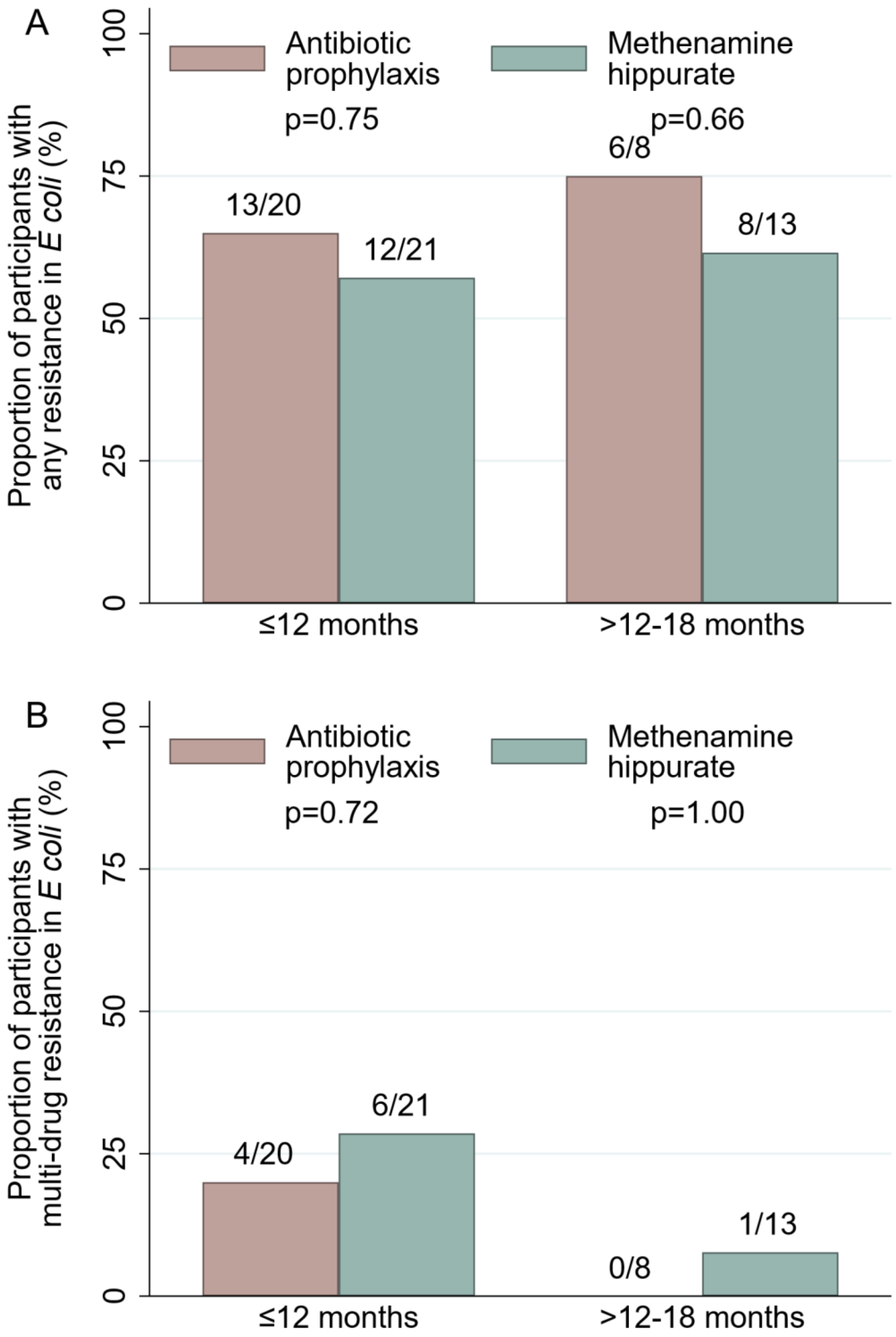

(A) Proportion of participants demonstrating resistance to at least one antibiotic in *E. coli* isolated from a symptomatic urine sample (out of those with *E. coli* isolated from a symptomatic urine sample). χ^2 p-values. (B) Proportion of participants demonstrating multi-drug resistance in *E. coli* isolated from a symptomatic urine samples (of those with *E. coli* isolated from a symptomatic urine sample). Fisher's exact p-values.

Figure s3: Antibiotic resistance rates (per sample) in *E. coli* isolated from symptomatic urine samples submitted during the 12-month preventive treatment period and 6-month post-treatment observational period

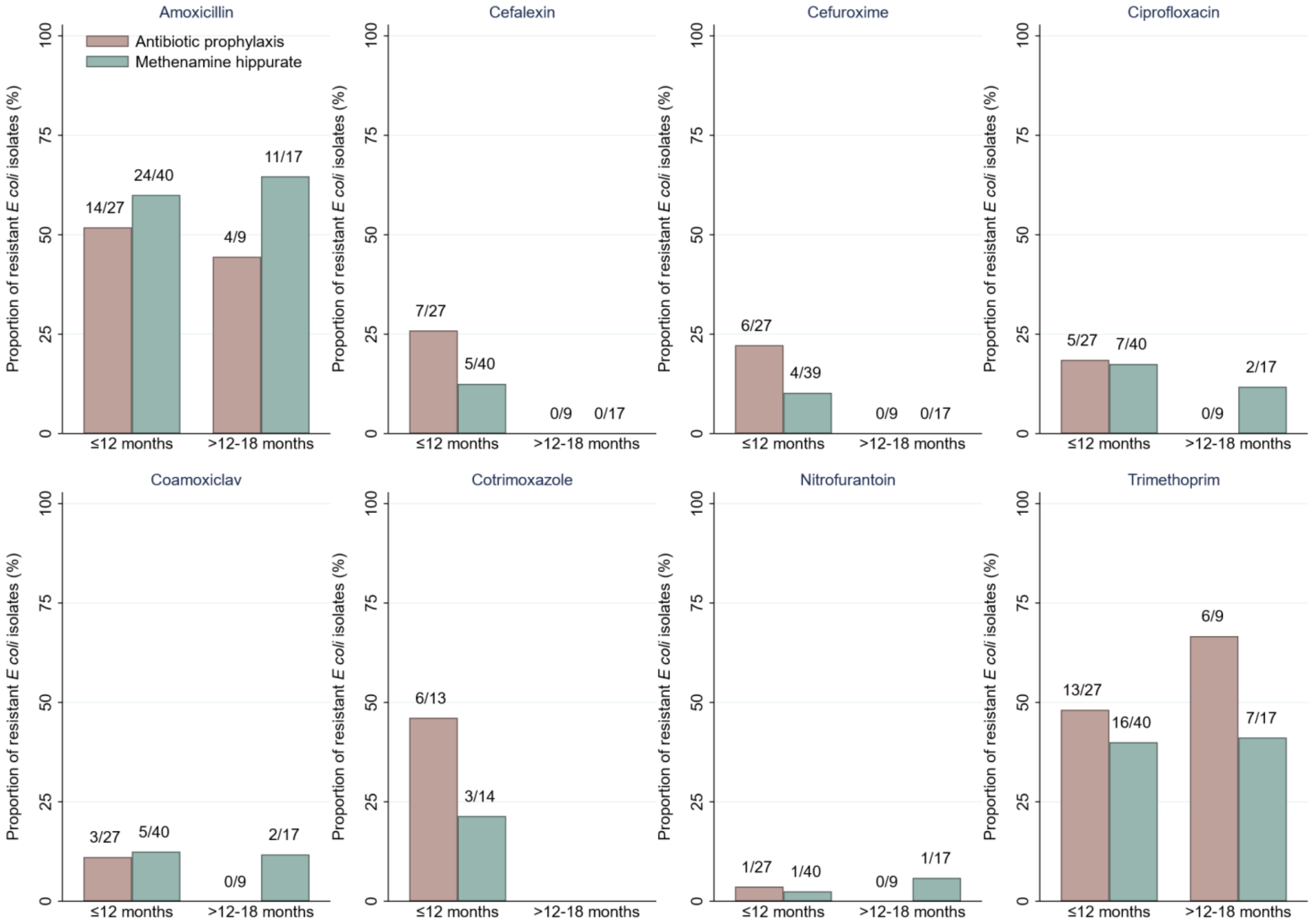

Table s10: Treatment satisfaction questionnaire for medication (TSQM) domain scores

	Antibiotic Prophylaxis (N=120)	Methenamine hippurate (N=120)	Comparison between groups	
			Mean difference (95% CI); two-sample t-test, p-value	ANCOVA‡, p-value
**Month 12**				
Effectiveness	80·0 (22·5); 63	77·5 (23·7); 74	-2·5 (-10·4, 5·4); 0·53	0·43
Side effects	92·9 (19·2); 62	95·8 (13·7); 72	2·9 (-2·7, 8·5); 0·31	0·29
Convenience	91·4 (12·7); 64	82·2 (18·4); 73	-9·2 (-14·6, -3·8); 0·001	0·001
Global satisfaction	80·6 (22·4); 64	77·3 (23·9); 73	-3.3 (-11·2, 4·5); 0·40	0·34
**Month 18**				
Effectiveness	74·4 (28·8); 57	75·0 (24·7); 69	0·7 (-8·8, 10·1); 0·89	0·95
Side effects	93·2 (18·2); 55	94·9 (15·7); 70	1·7 (-4·3, 7·7); 0·57	0·56
Convenience	85·7 (17·0); 59	84·6 (15·7); 72	-1·1 (-6·8, 4·5); 0·69	0·70
Global satisfaction	75·8 (25·5); 60	74·7 (27·1); 72	-1·1 (-10·3, 8·0); 0·81	0·70

Data are mean (SD); number with data unless otherwise specified. Possible scores range from 0-100 with higher scores indicating increased satisfaction. ‡ Adjusted for menopausal status (pre and peri/post) and prior UTI frequency (<4 and ≥4).

Figure s4: Box plots of kidney and liver function blood tests over time, by randomised treatment group

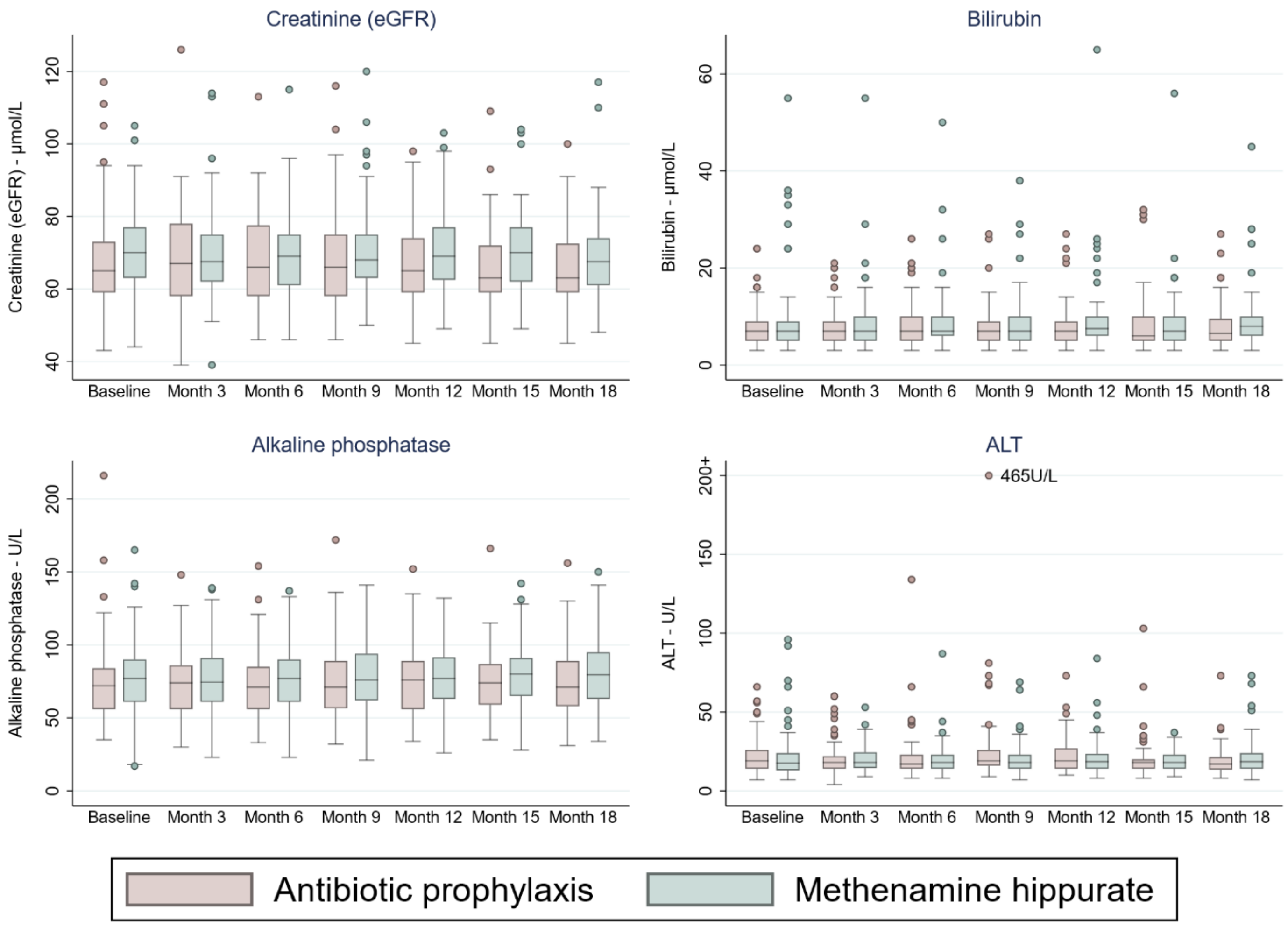

ALT: Alanine transaminase
